# Emergence of *Raoultella ornithinolytica* isolated from chicken products in Alexandria, Egypt

**DOI:** 10.14202/vetworld.2020.1473-1479

**Published:** 2020-07-29

**Authors:** Sara M. El-Shannat, Ashraf A. Abd El-Tawab, Wafaa M. M. Hassan

**Affiliations:** 1 Department of Microbiology, Animal Health Research Institute, Marsa Matruh, Egypt; 2 Department of Bacteriology, Immunology, and Mycology, Faculty of Veterinary Medicine, Benha University, Banha, Egypt; 3 The Reference Laboratory for Quality Control on Poultry Production, Animal Health Research Institute, Dokki, Giza, Egypt

**Keywords:** analytical profile index 20E, matrix-assisted laser-desorption ionization time-of-flight mass spectrometry, phenotypic system, *Raoultella ornithinolytica*

## Abstract

**Background and Aim:**

*Raoultella ornithinolytica* is one of the emerging gram-negative bacteria, which associated with foodborne illness. Researches affirmed that distinguish between *R. ornithinolytica* and *Klebsiella oxytoca* are difficult, as they are phylogenetic related. The evolution of multidrug resistance of *Raoultella* strains gained more concern for recognition of the pathogen which supports in controlling the disease and minify its threat. This study sought to find a reliable tool for the identification of *Raoultella ornithinolytica*, isolated from chicken product samples, and assessed the resistance profile of *R*. *ornithinolytica* using antibiogram sensitivity tests.

**Materials and Methods:**

Forty samples of chicken products were collected between January and September 2019 from different markets in Alexandria Governorate, Egypt. The products included nuggets, strips, burgers, luncheon meats, pane, frankfurters, and minced chicken meat. The samples were transferred to the Reference Laboratory. The samples were subjected to isolation, biochemical reaction testing, phenotypic system analytical profile index (API) E20, and a detection of antimicrobial susceptibility test. Phenotypic identification was confirmed through matrix-assisted laser-desorption ionization time-of-flight mass spectrometry (MALDI-TOF MS).

**Results:**

Thirty-three bacterial isolates (82.50%) out of 40 samples were isolated into pure cultures from the chicken samples. Three isolates (9.09%) were positive for *R. ornithinolytica*, while 30 isolates (90.91%) exhibited growth characters for different pathogens (*Escherichia coli, Enterobacter aerogenes*, *Proteus vulgaris*, *R. ornithinolytica*, and *Klebsiella pneumoniae*). The isolates of *R. ornithinolytica* were resistant to five types of antibiotics and sensitive to two types of antibiotics.

**Conclusion:**

This study reported the first case of *R. ornithinolytica* found in chicken products in Egypt. Phenotypic system API 20E and MALDI-TOF MS were found to be reliable tools for confirming the diagnosis of *R. ornithinolytic*a. As it provides rapid identification with high sensitivity and specificity for *R. ornithinolytica*, which often do not require a molecular procedure for confirmation.

## Introduction

*Raoultella ornithinolytica* is recognized as histamine-producing Gram-negative bacteria, which usually inhabits aquatic environments such as saturated soil, water systems, and fish [1-6]. These bacteria belong to *Enterobacteriaceae* family, with distinctive biochemical characteristics that can assist with their discrimination from other phenotypically related species [2,7-11]. It has low nutritional demands and has the ability to survive when food is scarce. Moreover, the growth temperature required is remarkably variable from 4°C to 40°C [1]. In the past, *R. ornithinolytica* was classified as a member of *Klebsiella* genus and reclassified in 2001 as a new genus *Raoultella*, based on the sequencing of the 16S rRNA and the rpoB gene [1,12].

The use of a standard classical approach is considered a useful tool in the identification of *R. ornithinolytica* [13,14]. However, matrix-assisted laser-desorption ionization time-of-flight mass spectrometry (MALDI-TOF MS) and sequence analysis are more highly regarded as they offer the potential for extraordinary insight into pathogens [15].

This study sought to find a reliable tool for the identification of *R. ornithinolytica*, isolated from chicken product samples, and then assessed the resistance profile of *R*. *ornithinolytica* using antibiogram sensitivity tests.

## Materials and Methods

### Ethical approval

No ethical approval was needed to perform this study. However, the samples were treated according to the national and international criteria.

### Study duration, location, and data collection

This study took place from January 2019 to September 2019. Forty samples of chicken products were collected from different markets located in or near the center of Alexandria city. The products included nuggets, strips, burgers, luncheon meats, pane, frankfurters, and minced chicken meat. Samples were transported on ice to the Reference Laboratory for Quality Control on Poultry Production, Animal Health Research Institute, Dokki, Giza, Egypt. These samples were sealed in sterile bags and stored at −86°C until testing.

### Bacteriological examination of *R. ornithinolytica*

The samples were pretreated according to a method described previously [16]; briefly, samples were thawed at room temperature, macerated into small pieces using sterile blades, and homogenized using a sterile mortar and pestle. The samples were incubated under aerobic conditions using pre-enrichment (buffered peptone water) at 37°C for 24 h (HiMedia^®^, India). Twenty-five grams of the chicken product sample were pre-enriched into 225 ml buffered peptone water. Then, 0.1 ml and 1 ml of pre-enriched aliquots were transferred into 10 ml Rappaport and Vassiliadis broth for the enrichment and then incubated at 42°C for 24 h (HiMedia^®^, India). The enriched aliquot samples were seeded onto MacConkey and Xylose Lysine Deoxycholate agar (XLD) and incubated at 37°C for 24–48 h to observe colonial growth (HiMedia^®^, India) [17].

### Recommended biochemical tests panel for the identification of *R. ornithinolytica*

The four biochemical tests utilized are illustrated in Table-1 to provide an example of the test variability that occurs using the standard laboratory methods for each test since complete standardization of these biochemical methods has not yet been fully elucidated. Thus, some reports may not provide sufficient data [7,8,16].

**Table-1:** Biochemical identification for *R. ornithinolytica.*

Sample no.	Urease	Oxidase	T.S.I			L.I.A			
	
			Butt/G	Slant	H_2_S	Slant	Butt	G	H_2_S
16	weak +ve	−ve	AG	A	−ve	K^(P)^	A	−ve	−ve
28	+ve	−ve	AG	A	+ve (black PPt)	K^(P)^	A	−ve	−ve
40	+ve	−ve	AG	A	+ve (black PPt)	K^(P)^	A	−ve	−ve

P (remain purple or the original color), A (acid), K (alkaline), and G (gas).* R. ornithinolytica=Raoultella ornithinolytica*

### Identification using a phenotypic system analytical profile index (API) 20E strips

Phenotypic system API 20E strips are considered a well-established method for accurate identification, this method depends on the standardized extensive databases [18], the selected isolates and reference strains were then evaluated using a (identification of products) testing kit API 20E (bioMérieux) to minimize the misidentified results of *R. ornithinolytica* strains with *Klebsiella oxytoca* using a conventional laboratory technique. Preparation procedures occurred based on the manufacturer’s recommendation protocol, and the results were analyzed using the API 20E WEB™ service.

### Identification of *R. ornithinolytica* by MALDI-TOF MS fingerprinting

MALDI-TOF MS fingerprinting (Brüker Daltonik, GmbH, Bremen, Germany; Biotyper 3.0 database) was performed according to the following steps. The samples were prepared based on the manufacturer’s guidelines for the identification of Gram-negative bacteria using a formic acid method; the fresh isolates were inoculated into spots on the target plate. Then, 1 μl of 70% formic acid was placed in the microbial spot and left to dry at room temperature. Thereafter, 1 μl of MALDI matrix solution was added (saturated α-cyano-4-hydroxycinnamic acid in 50% acetonitrile and 2.5% trifluoroacetic acid) [19]. Data analysis of MALDI-TOF MS required a fingerprint comparison with a database of reference spectra according to the manufacturer’s recommendations through the use of various algorithms.

### Antibiotic susceptibility testing

*R. ornithinolytica* isolates were subjected to antibiotic susceptibility tests according to methods described and interpreted by Clinical and Laboratory Standards Institute (CLSI), 2018. The diffusion disks used in this study were obtained (Oxoid, U.K) as standard reference disks with known potency for laboratory use including flucloxacillin (FLX) 5 μg; nalidixic acid (NA) 30 μg; ciprofloxacin (CIP) 5 μg; oxytetracycline (OTC) 30 μg; tetracycline (TE) 30 μg; amikacin (AMK) 30 μg; streptomycin (S) 10 μg; erythromycin (ERY) 15 μg; bacitracin (BAC) 10 μg; colistin (CS) 10 μg; chloramphenicol (C) 30 μg; amoxicillin (AMX) 25 μg; AMX with clavulanic acid (AMC) 30 μg; ampicillin (AMP) 10 μg; and penicillin (P) 10 IU. All *R. ornithinolytica* isolates were subjected to a disk diffusion test. A suspension of each isolate was created with turbidity at 0.5 McFarland standard andthen plated onto Mueller-Hinton agar plates (about 25-30 ml per 90mm plate, the depth of the medium was 4 mm) http://www.uphs.upenn.edu/bugdrug/antibiotic_manual/bk.html. Antibiotic sensitivity disks were applied to each plate, and the plates were incubated at 37°C for 24 h. Then, the zones of inhibition were measured.

The antibiotic susceptibilities of *R. ornithinolytica* isolates were also determined and interpreted in accordance with CLSI, 2018. Briefly, the samples were subjected to 15 distinct antibiotics (FLX, NA, CIP, OTC, TE, AMK, S, ERY, BAC, CS, C, AMX, AMC, AMP, and P) (Table-2), at a minimum inhibitory concentration and the breakpoint was 5 μg, 30 μg, 5 μg, 30 μg, 30 μg, 30 μg, 10 μg,15 μg,10 μg, 10 μg, 30 μg, 25 μg, 30 μg, 10 μg, and 10 IU, respectively (Table-2). Antibiotic sensitivity disks were applied to each prepared plate and the plates were incubated at 37°C for 24 h. Then, the zones of inhibition were measured.

**Table-2:** Result of disk susceptibility determinations for *R. ornithinolytica* samples.

No.	*Antibiotics*	Conc./MG	Sample 16		Sample 28		Sample 40	
		
			Zone diam. (mm)	Interpretation	Zone diam. (mm)	Interpretation	Zone diam. (mm)	Interpretation
1	FLX	5 μg	6	R	6	R	6	R
2	NA	30 μg	25	S	16	I	20	S
3	CIP	5 μg	30	S	22	S	20	I
4	OTC	30 μg	20	S	6	R	6	R
5	TE	30 μg	21	S	6	R	6	R
6	AMK	30 μg	19	S	18	S	16	I
7	S	10 μg	16	S	6	R	6	R
8	ERY	15 μg	6	R	6	R	6	R
9	BAC	10 μg	6	R	6	R	6	R
10	CS	10 μg	13	S	12	S	12	S
11	C	30 μg	26	S	25	S	26	S
12	AMX	25 μg	16	I	6	R	6	R
13	AMC	30 μg	11	R	6	R	10	R
14	AMP	10 μg	13	I	6	R	6	R
15	P	10 IU	6	R	6	R	6	R

FLX=Flucloxacillin, NA=Nalidixic acid, CIP=Ciprofloxacin, OTC=Oxytetracycline, TE=Tetracycline, AMK=Amikacin, S=Streptomycin, ERY=Erythromycin, BAC=Bacitracin, CS=Colistin, C=Chloramphenicol, AMX=Amoxicillin, AMC=Amoxicillin with clavulanic acid, AMP=Ampicillin, and P=Penicillin. R=Resistance, I=Intermediate, and S=Sensitive

## Results

**Figure-1:**
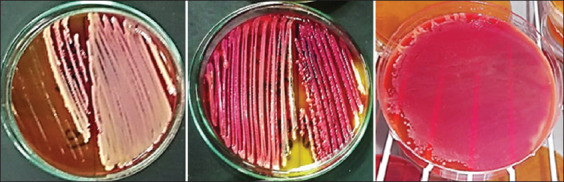
Growth of *Raoultella ornithinolytica* colonies on XLD agar.

Forty samples of chicken products were subjected to conventional identification. Data illustrated in Table-3 and Figure-1 show the results of sample cultivation on XLD, from the different locations in Alexandria Governorate, Egypt. As shown in Table-3, 33 bacteria were isolated and grown into pure cultures from the chicken product samples. The isolated bacteria consisted of 12.1% (4/33 isolates) *Escherichia coli*, 6.0% (2/33 isolates) *Klebsiella aerogenes*, 3.0% (1/33 isolates) *Proteus vulgaris*, 9.0% (3/33 isolates) *R. ornithinolytica*, and 69.6% (23/33 isolates) *K. pneumoniae*.

**Table-3:** Results of samples cultivation on Xylose Lysine Deoxycholate agar (XLD).

Sample no.	Collection area	Source of sample	Result of cultivation on XLD
1	Borg El Arab	Plain chicken luncheon	+ve
2	Ras at-Tin	Nuggets	+ve
3	Loran	Strips	+ve
4	Al Anfoshy	Spicy chicken pane	+ve
5	Ras at-Tin	Chicken burger	+ve
6**	Flemeg	Chicken burger	−ve
7**	Ras El-tin	Crunchy spicy chicken pane	−ve
8	Elanfoshy	Spicy chicken pane	+ve
9**	Ras at-Tin	Chicken burger	−ve
10	Kamp Shizar	Chicken burger	+ve
11	Borg El Arab	Chicken luncheon	+ve
12	El Raml	Chicken luncheon	+ve
13**	Al Ibrahimeyya	Crunchy spicy chicken pane	−ve
14	North coast	Chicken luncheon with olive	+ve
15	North coast	Chicken luncheon with olive	+ve
16*	Borg EL Arab	Plain chicken luncheon	+ve
17	Al-Ibrahimiyyah	Chicken fingers	+ve
18	El Ras El Souda	Chicken fingers	+ve
19	El Seyouf	Chicken frankfurter	+ve
20	El Ras el Souda	Chicken frankfurter	+ve
21	Sidi Bishr	Chicken frankfurter	+ve
22	Sidi Beshr	Chicken burger	+ve
23	Roshdy	Chicken burger	+ve
24	El Seyouf	Chicken burger	+ve
25**	El Mandara	Chicken fingers	−ve
26	Gleem	Chicken fingers	+ve
27**	Smouha	Nuggets	−ve
28*	El Seyouf	Nuggets	+ve
29	El Shatbi	Smoked chicken luncheon	+ve
30	El Asafra	Chicken luncheon with olive	+ve
31	El Mandara	Plain chicken luncheon	+ve
32	Stanley	Plain chicken luncheon	+ve
33	Miami	Plain chicken luncheon	+ve
34	Miami	Chicken frankfurter	+ve
35**	Stanley	Chicken frankfurter	−ve
36	El Asafra	Chicken frankfurter	+ve
37	Moustafa Kamel	Chicken frankfurter	+ve
38	Sporting	Chicken frankfurter	+ve
39	Kafr Abdo	Chicken frankfurter	+ve
40*	El Seyouf	Nuggets	+ve

* Positive *R. ornithinolytica* on XLD.

** The samples did not show any growth on XLD.* R. ornithinolytica*=*Raoultella ornithinolytica*

**Figure-2:**
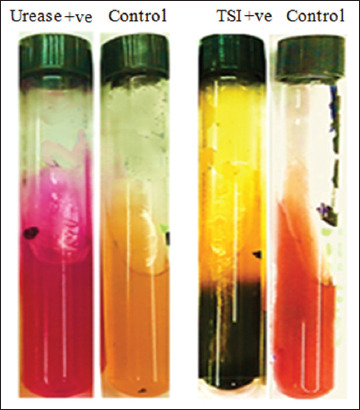
Identification of *Raoultella ornithinolytica* using Triple Sugar Iron Agar and urease test.

**Figure-3:**
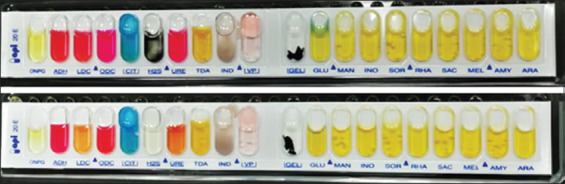
Identification of *Raoultella ornithinolytica* using phenotypic system API 20E for isolates no 28 and 40.

Table-1 and Figure-2 refer to the results of the individual biochemical reactions (oxidase, Triple Sugar Iron Agar (TSI), Lysine Iron Agar (LIA), and urease) for the expected positive *R. ornithinolytica* isolates, which were then identified as *R. ornithinolytica* by use of an API 20E test. The phenotypic system API 20E confirmed that the three pre-identified samples had proved to be *R. ornithinolytica* based on their positive results for ornithine decarboxylase, ODC+, which is the primary identification test that distinguishes between *R. ornithinolytic*a and *K. oxytoca* (Figure-3). Moreover, the three ODC+ samples were subjected to MALDI-TOF MS fingerprinting as a confirmation method for the results obtained by API 20E. MALDI-TOF MS verified the diagnosis of *R. ornithinolytica*. The obtained spectra were compared with a Biotyper database, which revealed that all three samples that were pre-identified using conventional techniques were 100% correctly identified as *R. ornithinolytica*, with a score value < 2.00. These results demonstrated that the most effective methods used for the identification of *R. ornithinolytica* were the phenotypic system API 20E combined with MALDI-TOF MS.

**Figure-4:**
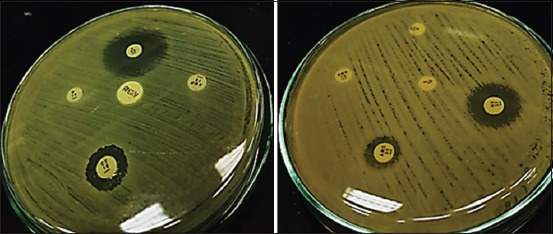
Mueller-Hinton agar media showing the susceptibility of 10 types of antibiotic against *Raoultella ornithinolytica* isolate (sample 40).

The three *R. ornithinolytica* samples were subjected to antibiotic susceptibility tests to evaluate their resistance profiles using antibiotic disks (Oxoid, U.K, depicted in Figure-4) [20,21]. The samples were exposed to 15 discrete antibiotics (Table-2), the first sample demonstrated resistance to five types of antibiotic disks (FLX, ERY, P, AMC, and BAC), intermediate sensitivity was found to two antibiotics (AMX and AMP), and sensitivity to eight antibiotics (C, CS, NA, CIP, TE, OTC, AMK, and S). The second sample demonstrated resistance to 10 antibiotics (FLX, AMP, ERY, P, TE, OTC, AMC, AMX, S, and BAC). Intermediate sensitivity was found to only one antibiotic (NA) and sensitive to four antibiotics (C, CIP, CS, and AMK). The third sample demonstrated resistance to 10 antibiotics (FLX, AMP, ERY, P, tetracycline, OTC, AMC, AMX, S, and BAC). Intermediate sensitivity was found for two antibiotics (CIP and AMK) and sensitivity to three antibiotics (C, CS, and NA).

## Discussion

In the past few decades, serious infections have occurred through emerging diseases. *R*. *ornithinolytica* infections were once rare in humans; however, recently, this pathogen is emerging and is associated with foodborne illness [22], toxicity, septicemia, bacteremia, enteric fever, scombroid poisoning, and immune deficiency [15,23-30]. Furthermore, *R*. *ornithinolytica* is commonly misidentified in clinical microbiology laboratories, as this bacterium is quite similar to *Klebsiella* species [31]. Therefore, the correct identification of *R*. o*rnithinolytica* is important for treating patients and improving the classification and characterization of this bacterial species. In the current study, three strains of *R. ornithinolytica* were isolated from chicken products (Figure-1), subjected to four manual laboratory biochemical tests, which included oxidase, TSI, LIA, and urease (Table-1). *R. ornithinolytica* was identified through the following morphological characters, biochemical reaction, growth temperature, and pigment production [7,8,16]. The first preliminary identification in this study was confused with *Klebsiella oxytoca*. Subsequent testing using the phenotype system API 20E (bioMérieux), identified these samples as *R. ornithinolytica*. API 20E (bioMérieux) is considered a discriminatory method which uses ornithine decarboxylase (ODC) tests to differentiate between *R. ornithinolytica* positive (ODC+) and *K. oxytoca* negative (ODC−) [18]. The phenotype recognition results were confirmed through an emerging microbial diagnostic technology (MALDI-TOF MS).

Then, the samples were subjected to disk diffusion to determine their antibiotic profiles. However, there are many variables that could influence the outcome of the data interpreted through disk susceptibility determinations, such as similarity in minimum inhibitory concentration for the antibiotic disk accompanied by great variation in the sensitivity result within the same species. A possible explanation for this conflict might be attributed to the fact that the resistance of *R. ornithinolytica* to different types of antibiotics was not increased sufficiently or might be due to a higher microbial load as reported in our study, there were some differences found in the susceptibility ratio between the parents of isolated strains varying from 1:4. Furthermore, the presence of more than 1 isolate from the same sample, taken at different times, showed variability in the susceptibility profile overtime [32].

The results of this study were consistent with that of other recent studies denoting the efficacy and efficiency of MALDI-TOF MS for the identification of Gram-negative bacteria [13,33,34]. Moreover, this study indicated that a replacement for the traditional identification method for *R. ornithinolytica* isolation in the microbiology laboratory was important. Since this technology was simple, fast, and reliable, API 20E and MALDI-TOF MS should be considered for further studies. However, improvements would be required in sample preparation and the availability of databases specifically designed to identify significant strains.

## Conclusion

This is the first study in Egypt that illustrates the isolation of the extremely rare pathogen *R. ornithinolytica* from the chicken products which demonstrated an excellent prognosis with antibiotic susceptibility, as reported in the literature.

## Authors’ Contributions

SME created the research and experimental design, performed the laboratory experiment, data analysis, and wrote the manuscript. AAAE supervised the experiment, checked the data analysis, and revised the manuscript. WMMH supervised the experiment, helped in the laboratory work, and revised the manuscript. All authors contributed to the drafting and revision of the manuscript. All authors read and approved the final manuscript.
